# Clinical description and mutational profile of a Moroccan series of patients with Rubinstein Taybi syndrome

**DOI:** 10.4314/ahs.v21i2.58

**Published:** 2021-06

**Authors:** Siham Chafai Elalaoui, Wiam Smaili, Julien Van-Gils, Patricia Fergelot, Ilham Ratbi, Mariam Tajir, Benoit Arveiler, Didier Lacombe, Abdelaziz Sefiani

**Affiliations:** 1 Génomique et Epidémiologie Moléculaire des Maladies Génétiques (G2MG), Centre GENOPATH, Faculté de Médecine et de Pharmacie, Université Mohammed V, Rabat, Maroc; 2 Département de Génétique Médicale, Institut National d'Hygiène, Rabat, Maroc; 3 CHU de Bordeaux, Service de Génétique Médicale, Hôpital Pellegrin, Bordeaux, France; 4 Université Bordeaux, Laboratoire Maladies Rares: Génétique et Métabolisme (MRGM), INSERM U1211, Bordeaux, France; 5 Faculté de Médecine et Pharmacie, Université Mohammed Premier, Oujda, Maroc

**Keywords:** Rubinstein Taybi syndrome, CREBBP gene, mutation, Moroccan

## Abstract

**Background:**

Rubinstein-Taybi syndrome (RSTS; OMIM 180849) is a rare autosomal dominant developmental disorder with an estimated prevalence of one case per 125,000 live births. RSTS is characterized by typical face, broad thumbs and halluces, short stature, and intellectual disability. Facial dysmorphy is characteristic with microcephaly, low frontal hairline, arched eyebrows, long eyelashes, convex profile of nose, narrow palate, and micrognathia. RSTS is mainly due to mutations or microdeletions of the CREBBP gene (about 60%) and more rarely of the EP300 gene (8%).

**Objective:**

Clinical description and identification of mutations of patients with Rubinstein Taybi syndrome

**Methods:**

PCR and direct sequencing of CREBBP gene.

**Results:**

We report here, the clinical and molecular data of a series of six Moroccan patients with a phenotype of RSTS. The molecular study of the major gene CREBBP (by Sanger Sequencing followed by CGH array, if sequence normal) revealed point mutations in five patients. For the sixth patient, CGH array revealed a microdeletion carrying the CREBBP gene. Through this work, we emphasize the importance of clinical expertise in the diagnosis, management and genetic counseling in Rubinstein Taybi syndrome.

## Introduction

Rubinstein-Taybi syndrome (RSTS; OMIM 180849, RSTS2; OMIM 613684) is a rare autosomal dominant genetic disease, with a prevalence estimated at one case per 125000 live births^1^. It is characterized by facial dysmorphy, microcephaly, broad thumbs and first toes, postnatal growth retardation, and intellectual disability.

Facial dysmorphy is characterized by low frontal hairline, arched eyebrows, downslanting palpebral fissures, hypertelorism, long eyelashes, a beaked nose, low set ears, arched palate, micrognathia, dental anomalies (overcrowding of teeth), and atypical smile (“grimacing”). Typically, hands present a big thumb and a clinodactyly of the fifth finger. Other skeletal anomalies are described, including ligamentous laxity, vertebral anomalies, and abducted thumbs^2^,^3^. Other features may include multiple malformations including cardiac, ocular, genitourinary, digestive, and/or cutaneous disorders. Constipation often presents throughout life and some patients are overweight in late childhood or early puberty. Tendency of overweight or obesity was reported in RSTS patients, especially during adolescence, needing specific growth charts for appropriate assessment of the growth^4^. The patients also have an increased risk of developing benign tumors (pilomatricomas) or malignant tumors (brain tumors, acute leukemias, lymphomas)^2^,^3^,^5^. RSTS patients have mild intellectual disability^6^,^7^,^8^. Patients have usually friendly and sociable characteristics. However, in some patients motor stereotypies, short attention span, and poor coordination have ben described^9^. Additionally, some RSTS had mood swings and obsessive compulsive behavior especially in adulthood6,10. More than 90% of RSTS patients survive to adulthood, but their healthcare is difficult, due to absence of standardized specific guidelines^10^.

Mutations in two highly conserved genes have been implicated in the etiology of RSTS. These are cAMP response element-binding protein (CREB) binding protein (CREBBP; NM_600140) localized at 16p13.3 and EA1-associated protein p300 (EP300; NM_602700) localized at 22q13 mutated respectively in about 60% and 8% of cases^11^,^12^. More than half of patients with RST have mutations in CREBBP gene. CREBBP and EP300 are two ubiquitous expression genes with a major role in chromatin remodeling by acetylation of histones, a mechanism particularly involved in neuronal plasticity and cognition^13^. There is no clear genotype phenotype correlation^14^. A few familial cases with germline and somatic mosaicism have been reported^15^,^16^,^17^. We report here a series of six Moroccan patients with a phenotype of RSTS.

## Case report

### Clinical report

Six patients with a facial dysmorphy and malformations presented to our Department of Medical Genetics. All the patients were examined by clinical geneticists and a suspicion of clinical diagnosis of RSTS was evoked for these patients. Informed consent was obtained for all the patients and their parents, and the study was performed with approval of the regional ethics committee at the National Institute of Health, Rabat. The Genomic DNA from the patients and their parents, when available, was isolated from peripheral blood according to the standard procedures. The Clinical data of the six patients is summarized in Table 1.

**Table 1:** Clinical and molecular findings of the six patients with Rubinstein Taybi Syndrome

Patients	Patient 1	Patient 2	Patient 3	Patient 4	Patient 5	Patient 6
Age at diagnosis	1 year	5 years	9 years	8 years	6 years	3 years
Sex	Male	Male	Female	Male	Male	Male
Growth retardation	+	+	+	+	+	+
Intellectual disability	+	+	+	+	+	+
Speech delay	+	+	+	+	+	+
Low anterior hairline	-	-	+	+	+	+
High arched ayebrows	+	+	+	+	+	+
Broad ayebrows	+	+	+	+	+	+
Long eyelashes	+	+	+	+	+	+
Down slanting palpebral fissures	+	+	+	+	+	+
Beaked nose	+	+	+	+	+	+
‘grimacing smile’,	+	+	+	+	-	-
Low set ears	+	+	+	+	+	+
Hisrsutism	+	-	+	+	+	+
Micrognathia	-	-	+	+	+	+
Broad thumbs	+	-	+	+	+	+
Angulated Thumbs	-	+	+	+	-	+
Broad halluces	+	-	+	+	+	+
Seizures	-	-	-	-	-	-
Urogenital malformation	+	+	-	+	-	+
Heart congenital malfromation	+	+	-	-	-	+
Other features	-	-	-	Club foot varus equinus	-	Feeding diffidculties
Mutation	c.3160G>T p.E1054X (nonsense)	c.6169C.T p.Q2057X (nonsense)	c.3609G.C p.K1203R (missense)	c.4350C>A p.Y1450X (nonsense)	c.3982+5G.A (splicing)	Deletion of a 100 kb including the exons 20 to 31 of *CREBBP* gene.
Chromosome genomic location of mutation	chr16:g.3767810 C>A	chr16:g.3728875 G>A	chr16:g.3757809 C>G	chr16:g.373860 3G>T	chr16:g.374488 9C>T	-
Inheritance	De novo	De novo	De novo	De novo	De novo	De novo

Patient 1 was the fifth child of healthy non consanguineous parents. He had no particular family history. The patient had normal gestation and delivery. He was followed for congenital heart defect. He had psychomotor delay. At last clinical examination at two years, he had hirsutism, synophris, convex nasal bridge, retrognathism, broad thumbs and halluces, and bilateral crytorchidism.

Patient 2 was the second child of healthy non consanguineous parents. There was no family history of malformations or intellectual disability. The birth weight was 2300 g. He had psychomotor delay and an important speech delay, with little spoken language. At last clinical examination at six years, he had high arched broad eyebrows. He had pulmonary stenosis and micropenis. He had delayed bone age, and his karyotype was normal 46,XY.

Patient 3 was the first child born to healthy non consanguineous parents. The gestation and delivery were without any problem. The girl had psychomotor delay. At last clinical examination at nine years, she had typical facies of RSTS with low frontal hairline, arched eyebrows, long eyelashes, downslanting palpebral fissures, columella below alae nasi, a highly arched palate, hirsutism, and broad thumbs and halluces.

Patient 4 was the fifth child of healthy non consanguineous parents. The boy was born at 39 weeks of gestation after a normal pregnancy. In neonatal period, he had hypotonia. He had psychomotor delay and speech delay. At last clinical examination at 9 years, he had low fontal hairline, arched eyebrows, long eyelashes, downslanting palpebral fissures, hirsutism, clinodactyly of fifth finger, bifid tongue, broad thumbs with radial angulation, broad halluces and pectus excavatum. He had also bilateral cryptorchidism, renal stones, and club foot varus equinus.

Patient 5 was the third child of healthy parents. He had psychomotor delay. At last clinical examination at 6 years, he had hirsutism, typical facies of RSTS, with broad thumbs and halluces.

Patient 6 was the only child of non consanguineous parents. The gestation and delivery were without any problem. He had psychomotor delay, and speech delay. At last clinical examination at four years, he had short neck, strabism, downslanting palpebral fissures, convexed nose, low set ears, high arched palate, broad thumbs and halluces, and cryptorchidism. He was followed for congenital heart defect (ventricular and atrial septum defects). He had also feeding difficulties, recurrent pulmonary infections. His karyotype was normal 46,XY.

The clinical phenotypes of the patients are summarized in Table 1. The patients displayed a classic RSTS phenotype with characteristic facial features, broad thumbs, and halluces and a variable degree of intellectual disability.

### Molecular studies

Informed consent was obtained for all patients and parents. Polymerase chain reaction (PCR) amplification, followed by direct sequencing of the coding sequence and the corresponding exon intron boundaries of CREBBP gene (GRCh38:CM000678.2). Sequencing of the coding regions was performed using Big Dye Terminator cycle sequencing kit 3.1 (Applied Biosystems, Foster City, CA) according to the manufacturer's standard protocol and sequenced on an ABI genetic analyzer. Search for gene dosage anomaly by targeted array Comparative Genome Hybridization (CGH): CREBBP and EP300 genes and the genomic regions of the bands 16p13.3 and 22q13.2 were studied using a custom high resolution chip on 60K Custom oligonucleotide slides (Agilent Technologies, Courtaboeuf, France). The average distance between two oligonucleotides is 100 bp in the genes of interest and a region of 50 kb on either side of these genes. Following the routine testing procedure, array comparative genomic hybridization was performed with the patient's DNA (Cyanin 5) against a control DNA (Cyanin 3) and analyzed by Agilent CytoGenomics 3.0.1.1 software.

Gene dosage anomaly was confirmed using semi Quantitative Multiplex Fluorescent Polymerase Chain Reaction (QMF-PCR).

## Results

“Five patients had causal point mutations in the CREBBP gene (NM_004380), comprising three nonsense mutations, one missense mutation, and one splice mutation. All variants were classified as Pathogenic or Likely Pathogenic according to the American College of Medical Genetics (ACMG) guidelines for classification of variants18. None of the variants were found in 123,136 exomes and 15,496 genomes from the Genome Aggregation Database (gnomAD, accessed March 2020, ACMG criterion PM2)19. Furthermore, all the mutations were found to be de novo after testing of both parents (ACMG criterion PS2). Three mutations were nonsense (ACMG criterion PVS1): c.3160G>T (p.E1054X), c.6169C>T (p.Q2057X), c.4350C>A (p.Y1450X) one splice site mutation (ACMG criterion PVS1): c.3982+5G>A, and one missense mutation c.3609G>C (p.K1203N).

**Figure 1:**
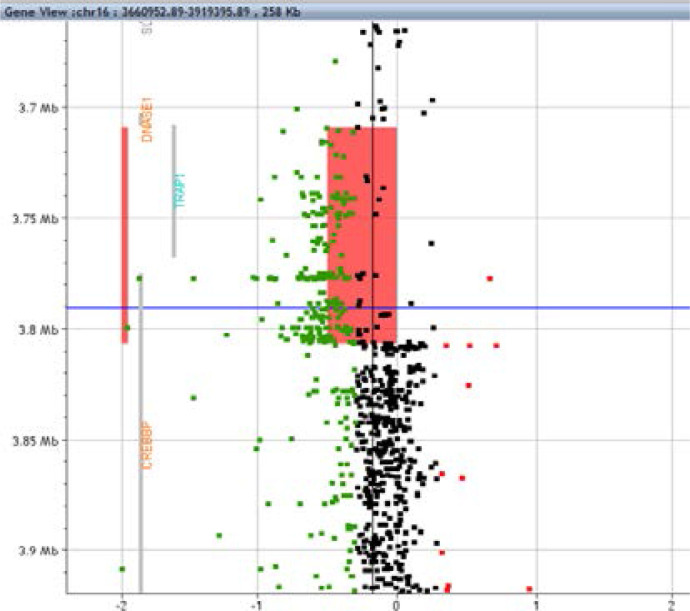
Custom array Comparative Genomic Hybridization (CGH). Targeted analysis at 16p13.3 showing the heterozygous deletion including the 3′end of CREBBP, the intergenic region and TRAP1 (red bar). Gene locations are indicated by grey bars. Gene view, Agilent Cytogenomics software V3, DLRS: 0,17, algorithm: ADM 2.6. Horizontal: log2ratio values (homogeneous deletion: -1), vertical: distance in megabases (Mb). The mosaic rate was estimated between 35 and 50% by comparing the mean deviation of all deleted probes (red rectangle) to the log2ratio values of non deleted probes (vertical black bar).

For the sixth patient, a heterozygous mosaic deletion was identified at 16p13.3 with a minimum size of 97,6 kb (hg19) and a maximum size of 100 kb: arr[ GRCh37]16p13.3(3708920- 3806511) x1. This deletion includes exons 20 to 31 of the CREBBP gene, and was expected to disrupt gene function (ACMG criterion PVS1). This deletion also includes the intergenic region and the entire TRAP1 gene (Figure 1). The rate of mosaicism has been estimated between 35 and 50% in leukocytes. All molecular findings of the six patients are summarized in table 1.”

## Discussion

Rubinstein-Taybi syndrome (RSTS) is a rare genetic syndrome and was first described in 19635. Its incidence is about 1 in 100,000 to 125,000 live births. Clinically, RSTS is characterized by postnatal intellectual disability, growth delay, microcephaly, dysmorphic facial features, and broad thumbs and halluces^20^,^21^. Mutations of the CREBBP gene are identified in 50-70% of RSTS patients, while mutations in the EP300 gene have been reported in 5-8% patients 12,22,23,24,25. The CREBBP and EP300 genes are ubiquitously expressed and are highlyomologous. CREBBP gene comprises 31 exons, encoding 2442 amino acid CREB-binding protein, whereas EP300 consists of 2415 amino acids^26^. CREBBP gene is a co-activator in cyclic-AMP-regulated gene expression. This gene is highly conserved, with 95% homology between human and mouse^27^,^28^. CREBBP has an important role in cell growth and development especially by regulating the transcriptional process. Since CREBBP is highly conserved, a mutation or a large deletion is likely to cause the full RSTS phenotype.

No evident genotype-phenotype correlations have been identified in RSTS. However, a lethal phenotype was reported in three patients with contiguous gene deletions including CREBBP gene^29^. Mosaic microdeletions of CREBBP have been recently reported with less severe involvement30. Milder RSTS phenotype has been reported in patients with missense mutations^7^. Seizures were reported to be more often frequent in RSTS patients with CREBBP mutations^14^. RSTS phenotype such as growth retardation and risk of seizures seems to be modified by CREBBP mutation^14^.

RSTS is mostly sporadic, and only a rare familial RSTS cases have been reported to date^15^,^16^,^17^. The recurrence risk of RSTS is low, but a prenatal diagnosis of RSTS should be proposed to parents due to gonadal mosaicism risk. Somatic mosacism, was reported in an unaffected father of a boy with RSTS and in a mildly affected father of three children with RSTS^16^,^17^. The monitoring of RSTS patients should include cardiac evaluation, dietary monitoring, and ophthalmologic evaluation^24^. With the emergence of Next Generation Sequencing, clinical spectrum of RSTS patients has widened. RSTS patients with EP300 mutations had less marked facial signs, and better cognitive development^12^. Menke et al., reported 11 patients with a de novo missense mutation in exons 30 and 31 of CREBBP gene, but without RSTS phenotype, but 31. This new condition was then known as Menke-Hennekam syndrome (MIM618332).

RSTS patients are more at risk for tumors especially medulloblastoma; diffuse large-cell B-cell lymphoma; breast cancer; non-small cell lung carcinoma; and colon carcinoma. No clear genotype-phenotype correlation became evident^32^.

In our cohort of six patients with typically clinical features of RSTS, five were found having mutations of CREBBP gene, and the patient 6 had a microdeletion found by CGH array, including exons 20 to 31 of CREBBP gene, with a mosaicism estimated between 35% and 50%. The six variants in CREBBP gene were not reported previously in the literature in other Rubinstein-Taybi patients.The last patient had a typical facial dysmorphy of RSTS, and a moderate intellectual disability due probably to the mosaicism. All patients showed a phenotype of RSTS including broad thumbs and big toes, but facial dysmorphy was not present in all patients. The phenotype of patient 2 was atypical from the classical RSTS phenotype, especially the pronounced speech delay, the distinctive nose, had some resemblance with floating Harbor syndrome. This patient didn't have broad thumbs or big toes, and nor hirsutism. These atypical features were reported in some RSTS patients with nonsense CREBBP mutations^33^,^34^. We here report the CREBBP mutation spectrum in a series of six Moroccan patients with a clinical diagnosis of RSTS. Our results confirm that mutations in CREBBP are the major cause of RSTS.

## Conclusion

Most cases of RSTS are sporadic. Familial forms are extremely rare. This is the first Moroccan cohort, relating the clinical and molecular findings. This will allow us to provide adequate genetic counseling to the families, and to manage adequately the patients.

## List of abbreviations

- RSTS: Rubinstein-Taybi syndrome.

- PCR: Polymerase chain reaction.
